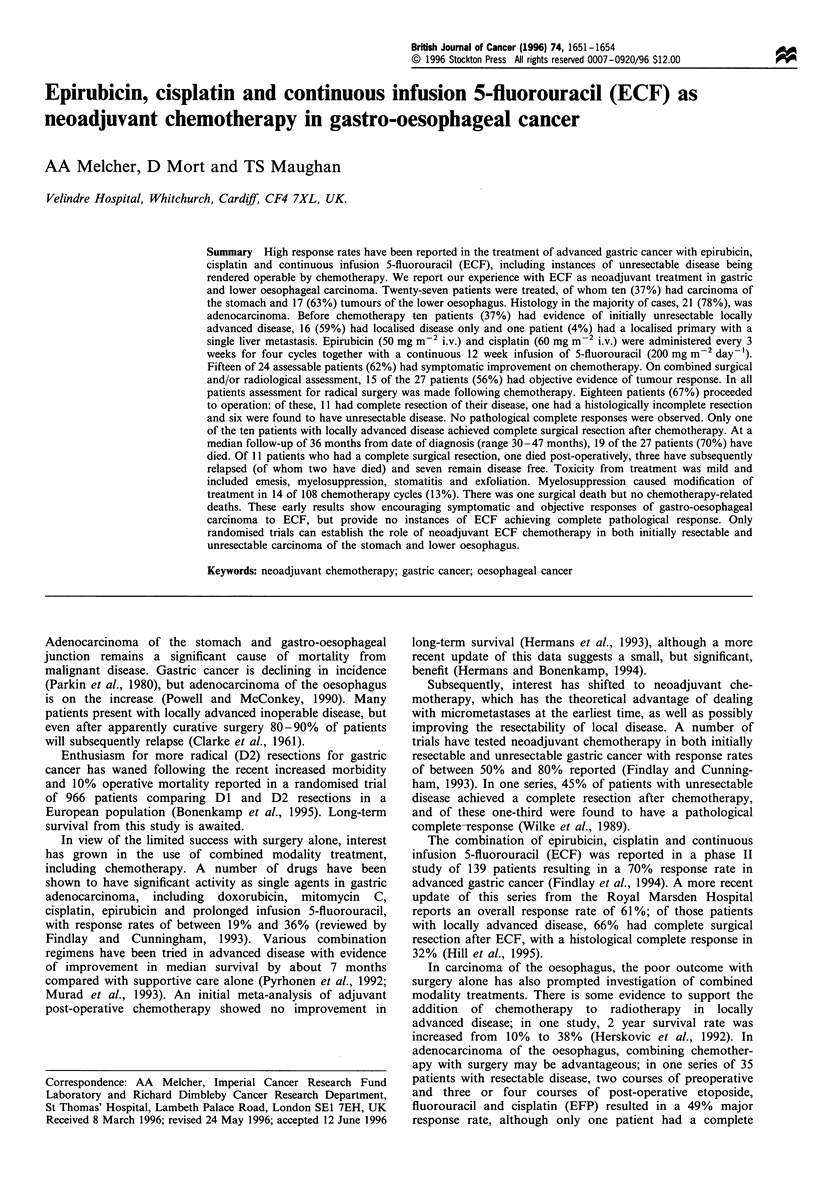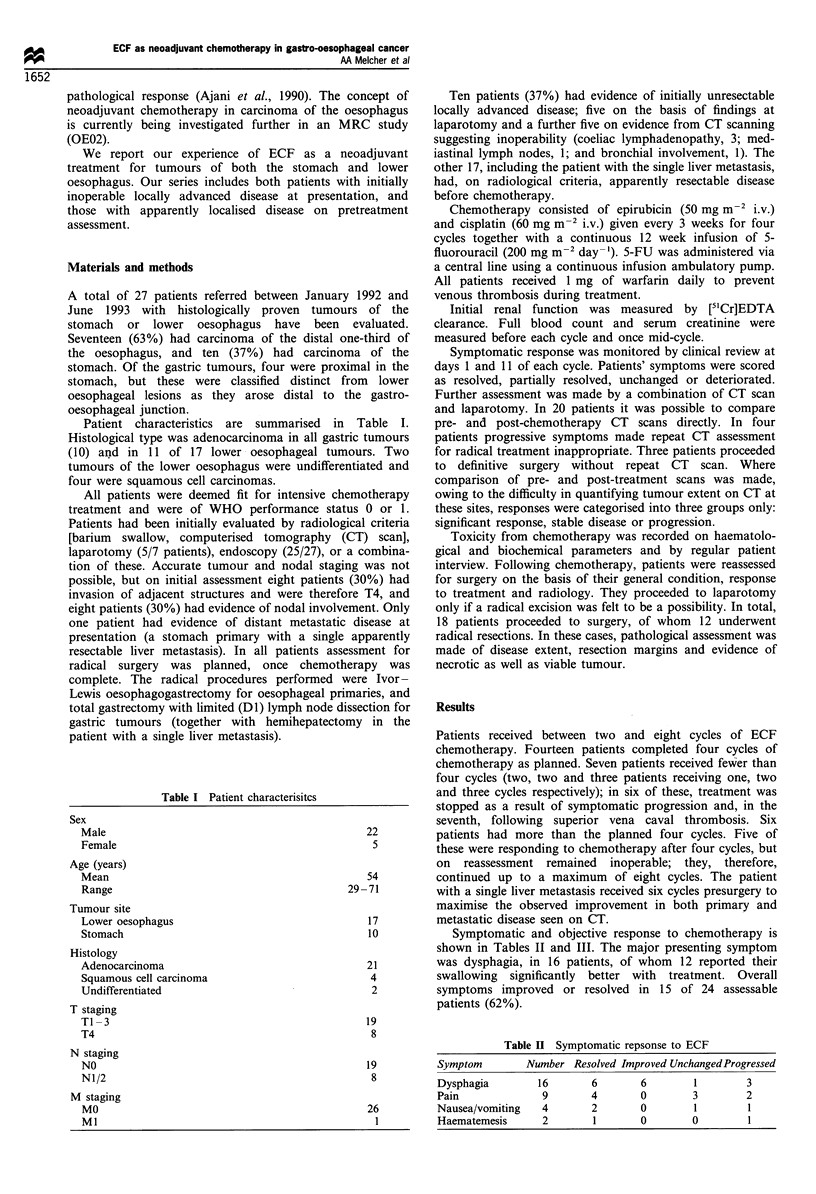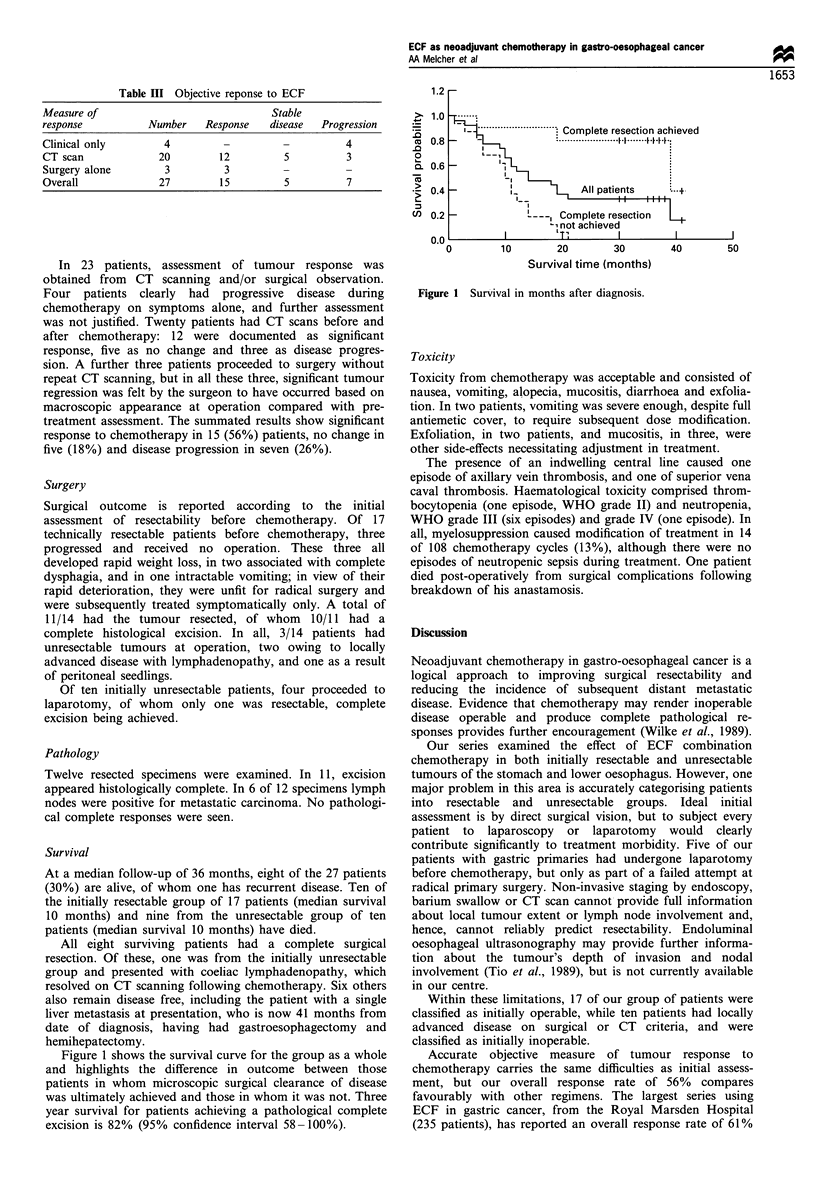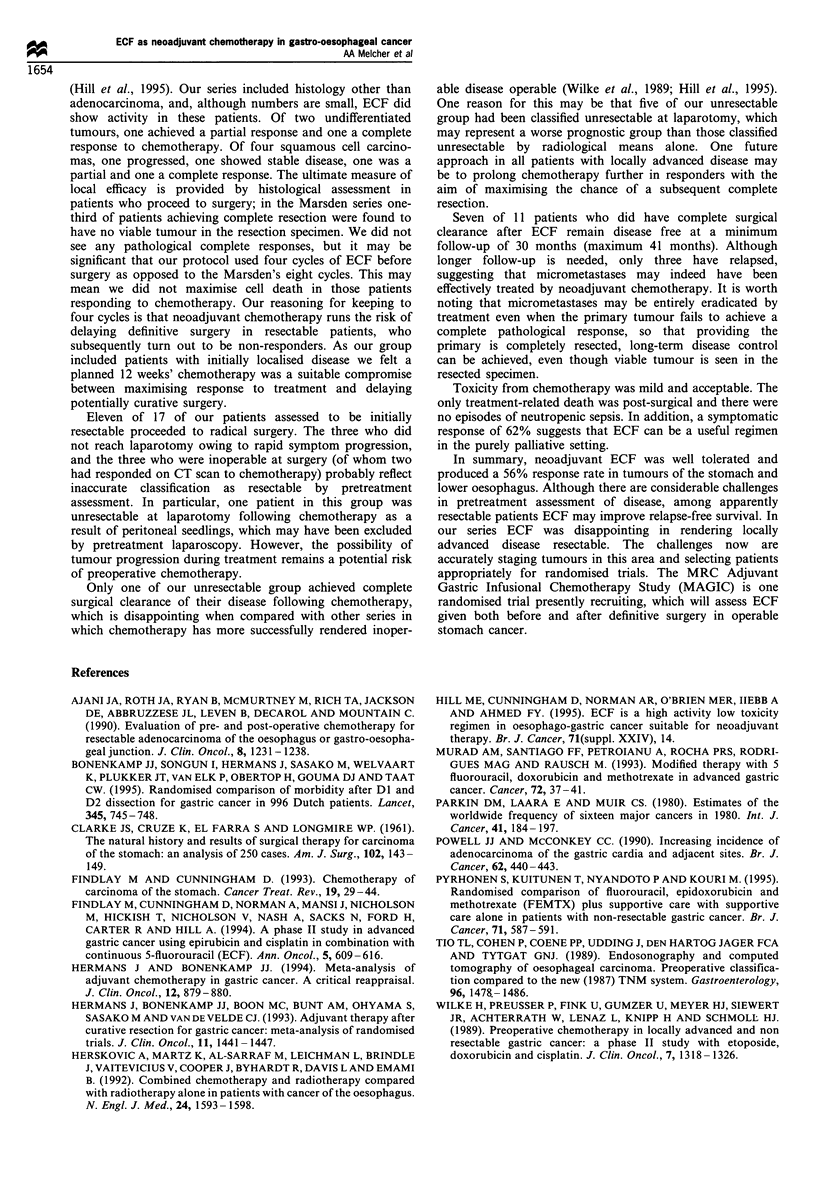# Epirubicin, cisplatin and continuous infusion 5-fluorouracil (ECF) as neoadjuvant chemotherapy in gastro-oesophageal cancer.

**DOI:** 10.1038/bjc.1996.604

**Published:** 1996-11

**Authors:** A. A. Melcher, D. Mort, T. S. Maughan

**Affiliations:** Velindre Hospital, Whitchurch, Cardiff, UK.

## Abstract

High response rates have been reported in the treatment of advanced gastric cancer with epirubicin, cisplatin and continuous infusion 5-fluorouracil (ECF), including instances of unresectable disease being rendered operable by chemotherapy. We report our experience with ECF as neoadjuvant treatment in gastric and lower oesophageal carcinoma. Twenty-seven patients were treated, of whom ten (37%) had carcinoma of the stomach and 17 (63%) tumours of the lower oesophagus. Histology in the majority of cases, 21 (78%), was adenocarcinoma. Before chemotherapy ten patients (37%) had evidence of initially unresectable locally advanced disease, 16 (59%) had localised disease only and one patient (4%) had a localised primary with a single liver metastasis. Epirubicin (50 mg m(-2) i.v.) and cisplatin (60 mg m(-2) i.v.) were administered every 3 weeks for four cycles together with a continuous 12 week infusion of 5-fluorouracil (200 mg m(-2) day(-1)). Fifteen of 24 assessable patients (62%) had symptomatic improvement on chemotherapy. On combined surgical and/or radiological assessment, 15 of the 27 patients (56%) had objective evidence of tumour response. In all patients assessment for radical surgery was made following chemotherapy. Eighteen patients (67%) proceeded to operation: of these, 11 had complete resection of their disease, one had a histologically incomplete resection and six were found to have unresectable disease. No pathological complete responses were observed. Only one of the ten patients with locally advanced disease achieved complete surgical resection after chemotherapy. At a median follow-up of 36 months from date of diagnosis (range 30-47 months), 19 of the 27 patients (70%) have died. Of 11 patients who had a complete surgical resection, one died post-operatively, three have subsequently relapsed (of whom two have died) and seven remain disease free. Toxicity from treatment was mild and included emesis, myelosuppression, stomatitis and exfoliation. Myelosuppression caused modification of treatment in 14 of 108 chemotherapy cycles (13%). There was one surgical death but no chemotherapy-related deaths. These early results show encouraging symptomatic and objective responses of gastro-oesophageal carcinoma to ECF, but provide no instances of ECF achieving complete pathological response. Only randomised trials can establish the role of neoadjuvant ECF chemotherapy in both initially resectable and unresectable carcinoma of the stomach and lower oesophagus.


British Journal of Cancer (1996) 74, 1651-1654
© 1996 Stockton Press All rights reserved 0007-0920/96 $12.00

### Epirubicin, cisplatin and continuous infusion 5-fluorouracil (ECF) as neoadjuvant chemotherapy in gastro-oesophageal cancer

AA Melcher, D Mort and TS Maughan

*Velindre Hospital, Whitchurch, Cardiff, CF4 7XL, UK.*

**Summary** High response rates have been reported in the treatment of advanced gastric cancer with epirubicin, cisplatin and continuous infusion 5-fluorouracil (ECF), including instances of unresectable disease being rendered operable by chemotherapy. We report our experience with ECF as neoadjuvant treatment in gastric and lower oesophageal carcinoma. Twenty-seven patients were treated, of whom ten (37%) had carcinoma of the stomach and 17 (63%) tumours of the lower oesophagus. Histology in the majority of cases, 21 (78%), was adenocarcinoma. Before chemotherapy ten patients (37%) had evidence of initially unresectable locally advanced disease, 16 (59%) had localised disease only and one patient (4%) had a localised primary with a single liver metastasis. Epirubicin (50 mg $m^{-2}$ i.v.) and cisplatin (60 mg $m^{-2}$ i.v.) were administered every 3 weeks for four cycles together with a continuous 12 week infusion of 5-fluorouracil (200 mg $m^{-2}$ $day^{-1}$). Fifteen of 24 assessable patients (62%) had symptomatic improvement on chemotherapy. On combined surgical and/or radiological assessment, 15 of the 27 patients (56%) had objective evidence of tumour response. In all patients assessment for radical surgery was made following chemotherapy. Eighteen patients (67%) proceeded to operation: of these, 11 had complete resection of their disease, one had a histologically incomplete resection and six were found to have unresectable disease. No pathological complete responses were observed. Only one of the ten patients with locally advanced disease achieved complete surgical resection after chemotherapy. At a median follow-up of 36 months from date of diagnosis (range 30-47 months), 19 of the 27 patients (70%) have died. Of 11 patients who had a complete surgical resection, one died post-operatively, three have subsequently relapsed (of whom two have died) and seven remain disease free. Toxicity from treatment was mild and included emesis, myelosuppression, stomatitis and exfoliation. Myelosuppression caused modification of treatment in 14 of 108 chemotherapy cycles (13%). There was one surgical death but no chemotherapy-related deaths. These early results show encouraging symptomatic and objective responses of gastro-oesophageal carcinoma to ECF, but provide no instances of ECF achieving complete pathological response. Only randomised trials can establish the role of neoadjuvant ECF chemotherapy in both initially resectable and unresectable carcinoma of the stomach and lower oesophagus.

**Keywords:** neoadjuvant chemotherapy; gastric cancer; oesophageal cancer

Adenocarcinoma of the stomach and gastro-oesophageal junction remains a significant cause of mortality from malignant disease. Gastric cancer is declining in incidence (Parkin *et al.*, 1980), but adenocarcinoma of the oesophagus is on the increase (Powell and McConkey, 1990). Many patients present with locally advanced inoperable disease, but even after apparently curative surgery 80-90% of patients will subsequently relapse (Clarke *et al.*, 1961).

Enthusiasm for more radical (D2) resections for gastric cancer has waned following the recent increased morbidity and 10% operative mortality reported in a randomised trial of 966 patients comparing D1 and D2 resections in a European population (Bonenkamp *et al.*, 1995). Long-term survival from this study is awaited.

In view of the limited success with surgery alone, interest has grown in the use of combined modality treatment, including chemotherapy. A number of drugs have been shown to have significant activity as single agents in gastric adenocarcinoma, including doxorubicin, mitomycin C, cisplatin, epirubicin and prolonged infusion 5-fluorouracil, with response rates of between 19% and 36% (reviewed by Findlay and Cunningham, 1993). Various combination regimens have been tried in advanced disease with evidence of improvement in median survival by about 7 months compared with supportive care alone (Pyrhonen *et al.*, 1992; Murad *et al.*, 1993). An initial meta-analysis of adjuvant post-operative chemotherapy showed no improvement in long-term survival (Hermans *et al.*, 1993), although a more recent update of this data suggests a small, but significant, benefit (Hermans and Bonenkamp, 1994).

Subsequently, interest has shifted to neoadjuvant chemotherapy, which has the theoretical advantage of dealing with micrometastases at the earliest time, as well as possibly improving the resectability of local disease. A number of trials have tested neoadjuvant chemotherapy in both initially resectable and unresectable gastric cancer with response rates of between 50% and 80% reported (Findlay and Cunningham, 1993). In one series, 45% of patients with unresectable disease achieved a complete resection after chemotherapy, and of these one-third were found to have a pathological complete response (Wilke *et al.*, 1989).

The combination of epirubicin, cisplatin and continuous infusion 5-fluorouracil (ECF) was reported in a phase II study of 139 patients resulting in a 70% response rate in advanced gastric cancer (Findlay *et al.*, 1994). A more recent update of this series from the Royal Marsden Hospital reports an overall response rate of 61%; of those patients with locally advanced disease, 66% had complete surgical resection after ECF, with a histological complete response in 32% (Hill *et al.*, 1995).

In carcinoma of the oesophagus, the poor outcome with surgery alone has also prompted investigation of combined modality treatments. There is some evidence to support the addition of chemotherapy to radiotherapy in locally advanced disease; in one study, 2 year survival rate was increased from 10% to 38% (Herskovic *et al.*, 1992). In adenocarcinoma of the oesophagus, combining chemotherapy with surgery may be advantageous; in one series of 35 patients with resectable disease, two courses of preoperative and three or four courses of post-operative etoposide, fluorouracil and cisplatin (EFP) resulted in a 49% major response rate, although only one patient had a complete

Correspondence: AA Melcher, Imperial Cancer Research Fund Laboratory and Richard Dimbleby Cancer Research Department, St Thomas' Hospital, Lambeth Palace Road, London SE1 7EH, UK
Received 8 March 1996; revised 24 May 1996; accepted 12 June 1996

pathological response (Ajani et al., 1990). The concept of neoadjuvant chemotherapy in carcinoma of the oesophagus is currently being investigated further in an MRC study (OE02).

We report our experience of ECF as a neoadjuvant treatment for tumours of both the stomach and lower oesophagus. Our series includes both patients with initially inoperable locally advanced disease at presentation, and those with apparently localised disease on pretreatment assessment.

### Materials and methods

A total of 27 patients referred between January 1992 and June 1993 with histologically proven tumours of the stomach or lower oesophagus have been evaluated. Seventeen (63%) had carcinoma of the distal one-third of the oesophagus, and ten (37%) had carcinoma of the stomach. Of the gastric tumours, four were proximal in the stomach, but these were classified distinct from lower oesophageal lesions as they arose distal to the gastro-oesophageal junction.

Patient characteristics are summarised in Table I. Histological type was adenocarcinoma in all gastric tumours (10) and in 11 of 17 lower oesophageal tumours. Two tumours of the lower oesophagus were undifferentiated and four were squamous cell carcinomas.

All patients were deemed fit for intensive chemotherapy treatment and were of WHO performance status 0 or 1. Patients had been initially evaluated by radiological criteria [barium swallow, computerised tomography (CT) scan], laparotomy (5/7 patients), endoscopy (25/27), or a combination of these. Accurate tumour and nodal staging was not possible, but on initial assessment eight patients (30%) had invasion of adjacent structures and were therefore T4, and eight patients (30%) had evidence of nodal involvement. Only one patient had evidence of distant metastatic disease at presentation (a stomach primary with a single apparently resectable liver metastasis). In all patients assessment for radical surgery was planned, once chemotherapy was complete. The radical procedures performed were Ivor-Lewis oesophagogastrectomy for oesophageal primaries, and total gastrectomy with limited (D1) lymph node dissection for gastric tumours (together with hemihepatectomy in the patient with a single liver metastasis).

**Table I** Patient characterisitcs

| | |
|---|---|
| Sex | |
| Male | 22 |
| Female | 5 |
| Age (years) | |
| Mean | 54 |
| Range | 29-71 |
| Tumour site | |
| Lower oesophagus | 17 |
| Stomach | 10 |
| Histology | |
| Adenocarcinoma | 21 |
| Squamous cell carcinoma | 4 |
| Undifferentiated | 2 |
| T staging | |
| T1-3 | 19 |
| T4 | 8 |
| N staging | |
| N0 | 19 |
| N1/2 | 8 |
| M staging | |
| M0 | 26 |
| M1 | 1 |

Ten patients (37%) had evidence of initially unresectable locally advanced disease; five on the basis of findings at laparotomy and a further five on evidence from CT scanning suggesting inoperability (coeliac lymphadenopathy, 3; mediastinal lymph nodes, 1; and bronchial involvement, 1). The other 17, including the patient with the single liver metastasis, had, on radiological criteria, apparently resectable disease before chemotherapy.

Chemotherapy consisted of epirubicin (50 mg $m^{-2}$ i.v.) and cisplatin (60 mg $m^{-2}$ i.v.) given every 3 weeks for four cycles together with a continuous 12 week infusion of 5-fluorouracil (200 mg $m^{-2}$ $day^{-1}$). 5-FU was administered via a central line using a continuous infusion ambulatory pump. All patients received 1 mg of warfarin daily to prevent venous thrombosis during treatment.

Initial renal function was measured by [$^{51}$Cr]EDTA clearance. Full blood count and serum creatinine were measured before each cycle and once mid-cycle.

Symptomatic response was monitored by clinical review at days 1 and 11 of each cycle. Patients' symptoms were scored as resolved, partially resolved, unchanged or deteriorated. Further assessment was made by a combination of CT scan and laparotomy. In 20 patients it was possible to compare pre- and post-chemotherapy CT scans directly. In four patients progressive symptoms made repeat CT assessment for radical treatment inappropriate. Three patients proceeded to definitive surgery without repeat CT scan. Where comparison of pre- and post-treatment scans was made, owing to the difficulty in quantifying tumour extent on CT at these sites, responses were categorised into three groups only: significant response, stable disease or progression.

Toxicity from chemotherapy was recorded on haematological and biochemical parameters and by regular patient interview. Following chemotherapy, patients were reassessed for surgery on the basis of their general condition, response to treatment and radiology. They proceeded to laparotomy only if a radical excision was felt to be a possibility. In total, 18 patients proceeded to surgery, of whom 12 underwent radical resections. In these cases, pathological assessment was made of disease extent, resection margins and evidence of necrotic as well as viable tumour.

### Results

Patients received between two and eight cycles of ECF chemotherapy. Fourteen patients completed four cycles of chemotherapy as planned. Seven patients received fewer than four cycles (two, two and three patients receiving one, two and three cycles respectively); in six of these, treatment was stopped as a result of symptomatic progression and, in the seventh, following superior vena caval thrombosis. Six patients had more than the planned four cycles. Five of these were responding to chemotherapy after four cycles, but on reassessment remained inoperable; they, therefore, continued up to a maximum of eight cycles. The patient with a single liver metastasis received six cycles presurgery to maximise the observed improvement in both primary and metastatic disease seen on CT.

Symptomatic and objective response to chemotherapy is shown in Tables II and III. The major presenting symptom was dysphagia, in 16 patients, of whom 12 reported their swallowing significantly better with treatment. Overall symptoms improved or resolved in 15 of 24 assessable patients (62%).

**Table II** Symptomatic repsonse to ECF

| *Symptom* | *Number* | *Resolved* | *Improved* | *Unchanged* | *Progressed* |
|---|---|---|---|---|---|
| Dysphagia | 16 | 6 | 6 | 1 | 3 |
| Pain | 9 | 4 | 0 | 3 | 2 |
| Nausea/vomiting | 4 | 2 | 0 | 1 | 1 |
| Haematemesis | 2 | 1 | 0 | 0 | 1 |

**Table III** Objective reponse to ECF

| *Measure of response* | *Number* | *Response* | *Stable disease* | *Progression* |
|---|---|---|---|---|
| Clinical only | 4 | – | – | 4 |
| CT scan | 20 | 12 | 5 | 3 |
| Surgery alone | 3 | 3 | – | – |
| Overall | 27 | 15 | 5 | 7 |

In 23 patients, assessment of tumour response was obtained from CT scanning and/or surgical observation. Four patients clearly had progressive disease during chemotherapy on symptoms alone, and further assessment was not justified. Twenty patients had CT scans before and after chemotherapy: 12 were documented as significant response, five as no change and three as disease progression. A further three patients proceeded to surgery without repeat CT scanning, but in all these three, significant tumour regression was felt by the surgeon to have occurred based on macroscopic appearance at operation compared with pre-treatment assessment. The summated results show significant response to chemotherapy in 15 (56%) patients, no change in five (18%) and disease progression in seven (26%).

*Surgery*

Surgical outcome is reported according to the initial assessment of resectability before chemotherapy. Of 17 technically resectable patients before chemotherapy, three progressed and received no operation. These three all developed rapid weight loss, in two associated with complete dysphagia, and in one intractable vomiting; in view of their rapid deterioration, they were unfit for radical surgery and were subsequently treated symptomatically only. A total of 11/14 had the tumour resected, of whom 10/11 had a complete histological excision. In all, 3/14 patients had unresectable tumours at operation, two owing to locally advanced disease with lymphadenopathy, and one as a result of peritoneal seedlings.

Of ten initially unresectable patients, four proceeded to laparotomy, of whom only one was resectable, complete excision being achieved.

*Pathology*

Twelve resected specimens were examined. In 11, excision appeared histologically complete. In 6 of 12 specimens lymph nodes were positive for metastatic carcinoma. No pathological complete responses were seen.

*Survival*

At a median follow-up of 36 months, eight of the 27 patients (30%) are alive, of whom one has recurrent disease. Ten of the initially resectable group of 17 patients (median survival 10 months) and nine from the unresectable group of ten patients (median survival 10 months) have died.

All eight surviving patients had a complete surgical resection. Of these, one was from the initially unresectable group and presented with coeliac lymphadenopathy, which resolved on CT scanning following chemotherapy. Six others also remain disease free, including the patient with a single liver metastasis at presentation, who is now 41 months from date of diagnosis, having had gastroesophagectomy and hemihepatectomy.

Figure 1 shows the survival curve for the group as a whole and highlights the difference in outcome between those patients in whom microscopic surgical clearance of disease was ultimately achieved and those in whom it was not. Three year survival for patients achieving a pathological complete excision is 82% (95% confidence interval 58–100%).

**Figure 1** Survival in months after diagnosis.

*Toxicity*

Toxicity from chemotherapy was acceptable and consisted of nausea, vomiting, alopecia, mucositis, diarrhoea and exfoliation. In two patients, vomiting was severe enough, despite full antiemetic cover, to require subsequent dose modification. Exfoliation, in two patients, and mucositis, in three, were other side-effects necessitating adjustment in treatment.

The presence of an indwelling central line caused one episode of axillary vein thrombosis, and one of superior vena caval thrombosis. Haematological toxicity comprised thrombocytopenia (one episode, WHO grade II) and neutropenia, WHO grade III (six episodes) and grade IV (one episode). In all, myelosuppression caused modification of treatment in 14 of 108 chemotherapy cycles (13%), although there were no episodes of neutropenic sepsis during treatment. One patient died post-operatively from surgical complications following breakdown of his anastamosis.

### Discussion

Neoadjuvant chemotherapy in gastro-oesophageal cancer is a logical approach to improving surgical resectability and reducing the incidence of subsequent distant metastatic disease. Evidence that chemotherapy may render inoperable disease operable and produce complete pathological responses provides further encouragement (Wilke *et al*., 1989).

Our series examined the effect of ECF combination chemotherapy in both initially resectable and unresectable tumours of the stomach and lower oesophagus. However, one major problem in this area is accurately categorising patients into resectable and unresectable groups. Ideal initial assessment is by direct surgical vision, but to subject every patient to laparoscopy or laparotomy would clearly contribute significantly to treatment morbidity. Five of our patients with gastric primaries had undergone laparotomy before chemotherapy, but only as part of a failed attempt at radical primary surgery. Non-invasive staging by endoscopy, barium swallow or CT scan cannot provide full information about local tumour extent or lymph node involvement and, hence, cannot reliably predict resectability. Endoluminal oesophageal ultrasonography may provide further information about the tumour's depth of invasion and nodal involvement (Tio *et al*., 1989), but is not currently available in our centre.

Within these limitations, 17 of our group of patients were classified as initially operable, while ten patients had locally advanced disease on surgical or CT criteria, and were classified as initially inoperable.

Accurate objective measure of tumour response to chemotherapy carries the same difficulties as initial assessment, but our overall response rate of 56% compares favourably with other regimens. The largest series using ECF in gastric cancer, from the Royal Marsden Hospital (235 patients), has reported an overall response rate of 61%

(Hill *et al.*, 1995). Our series included histology other than adenocarcinoma, and, although numbers are small, ECF did show activity in these patients. Of two undifferentiated tumours, one achieved a partial response and one a complete response to chemotherapy. Of four squamous cell carcinomas, one progressed, one showed stable disease, one was a partial and one a complete response. The ultimate measure of local efficacy is provided by histological assessment in patients who proceed to surgery; in the Marsden series one-third of patients achieving complete resection were found to have no viable tumour in the resection specimen. We did not see any pathological complete responses, but it may be significant that our protocol used four cycles of ECF before surgery as opposed to the Marsden's eight cycles. This may mean we did not maximise cell death in those patients responding to chemotherapy. Our reasoning for keeping to four cycles is that neoadjuvant chemotherapy runs the risk of delaying definitive surgery in resectable patients, who subsequently turn out to be non-responders. As our group included patients with initially localised disease we felt a planned 12 weeks' chemotherapy was a suitable compromise between maximising response to treatment and delaying potentially curative surgery.

Eleven of 17 of our patients assessed to be initially resectable proceeded to radical surgery. The three who did not reach laparotomy owing to rapid symptom progression, and the three who were inoperable at surgery (of whom two had responded on CT scan to chemotherapy) probably reflect inaccurate classification as resectable by pretreatment assessment. In particular, one patient in this group was unresectable at laparotomy following chemotherapy as a result of peritoneal seedlings, which may have been excluded by pretreatment laparoscopy. However, the possibility of tumour progression during treatment remains a potential risk of preoperative chemotherapy.

Only one of our unresectable group achieved complete surgical clearance of their disease following chemotherapy, which is disappointing when compared with other series in which chemotherapy has more successfully rendered inoperable disease operable (Wilke *et al.*, 1989; Hill *et al.*, 1995). One reason for this may be that five of our unresectable group had been classified unresectable at laparotomy, which may represent a worse prognostic group than those classified unresectable by radiological means alone. One future approach in all patients with locally advanced disease may be to prolong chemotherapy further in responders with the aim of maximising the chance of a subsequent complete resection.

Seven of 11 patients who did have complete surgical clearance after ECF remain disease free at a minimum follow-up of 30 months (maximum 41 months). Although longer follow-up is needed, only three have relapsed, suggesting that micrometastases may indeed have been effectively treated by neoadjuvant chemotherapy. It is worth noting that micrometastases may be entirely eradicated by treatment even when the primary tumour fails to achieve a complete pathological response, so that providing the primary is completely resected, long-term disease control can be achieved, even though viable tumour is seen in the resected specimen.

Toxicity from chemotherapy was mild and acceptable. The only treatment-related death was post-surgical and there were no episodes of neutropenic sepsis. In addition, a symptomatic response of 62% suggests that ECF can be a useful regimen in the purely palliative setting.

In summary, neoadjuvant ECF was well tolerated and produced a 56% response rate in tumours of the stomach and lower oesophagus. Although there are considerable challenges in pretreatment assessment of disease, among apparently resectable patients ECF may improve relapse-free survival. In our series ECF was disappointing in rendering locally advanced disease resectable. The challenges now are accurately staging tumours in this area and selecting patients appropriately for randomised trials. The MRC Adjuvant Gastric Infusional Chemotherapy Study (MAGIC) is one randomised trial presently recruiting, which will assess ECF given both before and after definitive surgery in operable stomach cancer.

### References

AJANI JA, ROTH JA, RYAN B, MCMURTNEY M, RICH TA, JACKSON DE, ABBRUZZESE JL, LEVEN B, DECAROL AND MOUNTAIN C. (1990). Evaluation of pre- and post-operative chemotherapy for resectable adenocarcinoma of the oesophagus or gastro-oesophageal junction. *J. Clin. Oncol.*, **8**, 1231–1238.

BONENKAMP JJ, SONGUN I, HERMANS J, SASAKO M, WELVAART K, PLUKKER JT, VAN ELK P, OBERTOP H, GOUMA DJ AND TAAT CW. (1995). Randomised comparison of morbidity after D1 and D2 dissection for gastric cancer in 996 Dutch patients. *Lancet*, **345**, 745–748.

CLARKE JS, CRUZE K, EL FARRA S AND LONGMIRE WP. (1961). The natural history and results of surgical therapy for carcinoma of the stomach: an analysis of 250 cases. *Am. J. Surg.*, **102**, 143–149.

FINDLAY M AND CUNNINGHAM D. (1993). Chemotherapy of carcinoma of the stomach. *Cancer Treat. Rev.*, **19**, 29–44.

FINDLAY M, CUNNINGHAM D, NORMAN A, MANSI J, NICHOLSON M, HICKISH T, NICHOLSON V, NASH A, SACKS N, FORD H, CARTER R AND HILL A. (1994). A phase II study in advanced gastric cancer using epirubicin and cisplatin in combination with continuous 5-fluorouracil (ECF). *Ann. Oncol.*, **5**, 609–616.

HERMANS J AND BONENKAMP JJ. (1994). Meta-analysis of adjuvant chemotherapy in gastric cancer. A critical reappraisal. *J. Clin. Oncol.*, **12**, 879–880.

HERMANS J, BONENKAMP JJ, BOON MC, BUNT AM, OHYAMA S, SASAKO M AND VAN DE VELDE CJ. (1993). Adjuvant therapy after curative resection for gastric cancer: meta-analysis of randomised trials. *J. Clin. Oncol.*, **11**, 1441–1447.

HERSKOVIC A, MARTZ K, AL-SARRAF M, LEICHMAN L, BRINDLE J, VAITEVICIUS V, COOPER J, BYHARDT R, DAVIS L AND EMAMI B. (1992). Combined chemotherapy and radiotherapy compared with radiotherapy alone in patients with cancer of the oesophagus. *N. Engl. J. Med.*, **24**, 1593–1598.

HILL ME, CUNNINGHAM D, NORMAN AR, O'BRIEN MER, IIEBB A AND AHMED FY. (1995). ECF is a high activity low toxicity regimen in oesophago-gastric cancer suitable for neoadjuvant therapy. *Br. J. Cancer*, **71**(suppl. XXIV), 14.

MURAD AM, SANTIAGO FF, PETROIANU A, ROCHA PRS, RODRIGUES MAG AND RAUSCH M. (1993). Modified therapy with 5 fluorouracil, doxorubicin and methotrexate in advanced gastric cancer. *Cancer*, **72**, 37–41.

PARKIN DM, LAARA E AND MUIR CS. (1980). Estimates of the worldwide frequency of sixteen major cancers in 1980. *Int. J. Cancer*, **41**, 184–197.

POWELL JJ AND MCCONKEY CC. (1990). Increasing incidence of adenocarcinoma of the gastric cardia and adjacent sites. *Br. J. Cancer*, **62**, 440–443.

PYRHONEN S, KUITUNEN T, NYANDOTO P AND KOURI M. (1995). Randomised comparison of fluorouracil, epidoxorubicin and methotrexate (FEMTX) plus supportive care with supportive care alone in patients with non-resectable gastric cancer. *Br. J. Cancer*, **71**, 587–591.

TIO TL, COHEN P, COENE PP, UDDING J, DEN HARTOG JAGER FCA AND TYTGAT GNJ. (1989). Endosonography and computed tomography of oesophageal carcinoma. Preoperative classification compared to the new (1987) TNM system. *Gastroenterology*, **96**, 1478–1486.

WILKE H, PREUSSER P, FINK U, GUMZER U, MEYER HJ, SIEWERT JR, ACHTERRATH W, LENAZ L, KNIPP H AND SCHMOLL HJ. (1989). Preoperative chemotherapy in locally advanced and non resectable gastric cancer: a phase II study with etoposide, doxorubicin and cisplatin. *J. Clin. Oncol.*, **7**, 1318–1326.